# Some Recent Contributions to Eugenics

**Published:** 1914-09

**Authors:** 


### The Department of Eugenics, Mental and Social Hygiene

CONDUCTED BY
DR. MALONE DUGGAN
813-814 Gibbs Building, San Antonio, Texas
All communications for this department should be sent to DR. DUGGAN

### Some Recent Contributions to Eugenics.

Prof. Wm. Bateson, one of the foremost exponents of the Mendelian theory and President of the British Association for the Advancement of Science, in a recent address at Melbourne on heredity, brought out some very important questions on this much mooted subject.

In speaking of variations, he stated that the old view of variations in form of life proceeding from the simple to the complex might be completely turned about, and that the true process of evolution might be from the complex to the simple.

He somewhat contradicts Darwin's idea that the temporary variations in the forms of life have come about wholly by crossing, and thus by the addition of new factors of life. He suggests the probability that the variations are due to the loss or suppression of factors present in the original species.

He said that "the doctrine of the survival of the fittest is undeniable so long as it is applied to the organism as a whole, but to attempt by this principle to find value in all definiteness of parts and functions, and in the name of science to see fitness everywhere is mere eighteenth-century optimism; the doctrine of the survival of favored races is a truism, helping scarcely at all to account for the diversity of species."

"The appearance of contemporary variability proves to be an illusion. Variation from step to step in the sexes must occur either by the addition or by the loss of a factor. Now of the origin of new forms by loss there seems to be fairly clear evidence, but of the contemporary acquisition of any new factor, I see no rare examples which may be so interpreted. We are left with a picture of variation utterly different from that which we saw at first. Variations now stand out as a definite physiological event."

He declared as untenable the contention of Losty that the sole source of variation was by crossing. Speaking further, he said,

"Having in view these and other considerations which might be developed, I feel no reasonable doubt that, though we may have to forego a claim to variations by additions of factors, yet variation both by loss of factors and by fractionation of factors is a genuine phenomenon of contemporary nature. If, then, we have to dispense, as it seems likely, with any addition from without, we must begin seriously to consider whether the course of evolution can be at all reasonably represented as an unpacking of an original complex which contained within itself the whole range of diversity which living things present. I do not suggest that we should come to a judgment as to what is or is not probable in these respects."

Speaking further regarding evolution he stated "as we have got to recognize that there has been an evolution, that somehow or other the forms of life have risen from fewer forms, we may as well see whether we are limited to the old view that evolutionary progress is from the simple to the complex, and whether after all, it is conceivable that the process was the other way about * * * At first it may seem rank absurdity to suppose that the primodial form or forms of protoplasm could have contained complexity enough to produce the divers types of life. But is it easier to imagine that these powers could have been conveyed by extrinsic additions?"

"I have confidence that the artistic gifts of mankind will prove to be due not to something added to the make-up of an ordinary man, but to the absence of factors which in the normal person inhabit the development of these gifts."

In concluding he said, "In truth, it is not these wider aspects of genetics that are at present our chief concern. They will come in their time. The great advancements of science are made like those of evolution, not by imperceptible mass improvements, but by the sporadic birth of penetrator genius. The journeymen follow after him, widening and clearing up, as we are doing along the tract that Mendel found."

Major Leonard Darwin, President of the British Eugenics Education Society is a eugenist that restricts the use of the word, "Eugenics" to those agencies affecting the inborn characters of future generations, and the term "social reforms, for those benefits which may be passed on to prosterity in ways other than those attributable to true biological heredity."

In a recent address before this society he said in part:

EUTHENICS FACILITATES RACIAL PROGRESS.

"The philanthropist's efforts resulted in the more easily cured or reformed being separated out from those less amenable to environmental influences. As social reform proceeded, and as the unfit were thus more and more clearly marked out from the nation at large, the numbers to be considered with reference to eugenic reform would be proportionately diminished, and racial progress would thus be facilitated. Social reforms producing these eugenic by-products must be utilized, as for example the proposed changes in the treatment of the habitual criminal. Improvement in environment would, no doubt, cause a diminution in crime, but a remnant of habitual criminals would remain, whose strong natural tendencies, being subject to laws of natural inheritance, would infallibly tend to reappear in their descendants. To lessen their fertility seemed, therefore, within the scope of engenic reform. Crime had a marked tendency to run in families."

EUGENICS NECESSARY FOR PERMENANT REFORM.

"Individuals endowed with those natural qualities, mental or physical, which render assistance to crime more than ordinarily difficult, are often brought into bad surroundings, mental or physical; this bad environment reacted on them, dragging them down in body and mind, and this action and reaction continuing either in the individual or generation after generation, the final resultant of these forces often was a long series of short imprisonments. The aim of the social reformer was, when possible, to break the vicious circle by at once removing the link of bad environment; whilst the eugenist would at the same time also strive to strenghten the innate characters of the individuals composing the coming generations. This latter result might be obtained by selective breeding.

"A study of criminal family statistics surely must make the believer in environmental effects demand the segregation of the criminal parent, both to safeguard the lives of those children who have been borne into foul surroundings and to lessen the numbers of those children who would be born to face the perils thus arising. In short that seemed to be the right policy to adopt from whatever direction they approached this subject."

THE INDETERMINENT SENTENCE.

"Few had studied these questions with care had any doubt that habitual criminals ought to be detained for longer periods than at present, whilst every effort should be made to make that detention more curative in its effects.

"A reform much more easily obtainable, and one which the eugenist ought to endeavor to promote, would be the amendment of the prevention of crimes act in such manner as to make it more readily applicable to the man of many minor offences. This act could easily be amended so as to make it easier to increase the periods of detention of those thousands of unfortunate persons who possess defects of character which drove them whenever free to a life of crime and made them an intolerable nuisance to society—defects which would inevitably reappear to some extent in their descendants if they had any. If they could get the paramount racial duty which they owed to posterity incorporated as an essential part of the moral code of the nation, then they would be on the high road to success."

